# Endogenous tagging of Unc-13 reveals nanoscale reorganization at active zones during presynaptic homeostatic potentiation

**DOI:** 10.3389/fncel.2022.1074304

**Published:** 2022-12-14

**Authors:** Sven Dannhäuser, Achmed Mrestani, Florian Gundelach, Martin Pauli, Fabian Komma, Philip Kollmannsberger, Markus Sauer, Manfred Heckmann, Mila M. Paul

**Affiliations:** ^1^Department of Neurophysiology, Institute of Physiology, University of Würzburg, Würzburg, Germany; ^2^Department of Neurology, Leipzig University Medical Center, Leipzig, Germany; ^3^Division of General Biochemistry, Rudolf Schönheimer Institute of Biochemistry, Medical Faculty, Leipzig University, Leipzig, Germany; ^4^Center of Mental Health, Department of Psychiatry, Psychotherapy, and Psychosomatics, University Hospital of Würzburg, Würzburg, Germany; ^5^Center for Computational and Theoretical Biology, Julius-Maximilians-Universität Würzburg, Würzburg, Germany; ^6^Department of Biotechnology and Biophysics, Julius-Maximilians-Universität Würzburg, Würzburg, Germany; ^7^Department of Orthopedic Trauma, Hand, Plastic and Reconstructive Surgery, University Hospital of Würzburg, Würzburg, Germany

**Keywords:** active zone, Unc-13, MiMIC, presynaptic homeostasis, nanoarchitecture, localization microscopy, *d*STORM, HDBSCAN

## Abstract

**Introduction:**

Neurotransmitter release at presynaptic active zones (AZs) requires concerted protein interactions within a dense 3D nano-hemisphere. Among the complex protein meshwork the (M)unc-13 family member Unc-13 of *Drosophila melanogaster* is essential for docking of synaptic vesicles and transmitter release.

**Methods:**

We employ minos-mediated integration cassette (MiMIC)-based gene editing using GFSTF (EGFP-FlAsH-StrepII-TEV-3xFlag) to endogenously tag all annotated *Drosophila* Unc-13 isoforms enabling visualization of endogenous Unc-13 expression within the central and peripheral nervous system.

**Results and discussion:**

Electrophysiological characterization using two-electrode voltage clamp (TEVC) reveals that evoked and spontaneous synaptic transmission remain unaffected in *unc-13*^GFSTF^** 3rd instar larvae and acute presynaptic homeostatic potentiation (PHP) can be induced at control levels. Furthermore, multi-color structured-illumination shows precise co-localization of Unc-13^GFSTF^, Bruchpilot, and GluRIIA-receptor subunits within the synaptic mesoscale. Localization microscopy in combination with HDBSCAN algorithms detect Unc-13^GFSTF^ subclusters that move toward the AZ center during PHP with unaltered Unc-13^GFSTF^ protein levels.

## Introduction

Synapses enable fast and efficient signaling in combination with structural miniaturization (Atwood and Karunanithi, 2002; Chua et al., 2010; Neher and Brose, 2018). Neurotransmitter is released at presynaptic active zones (AZs) consisting of a conserved set of core proteins (Südhof, 2012). One key determinant of synaptic strength is the number of release sites *N* (Silva et al., 2021). The molecular identity of *N* and its dynamics are currently a topic of intense investigations and have been linked to the AZ protein (M)unc-13 (Neher, 2010; Sakamoto et al., 2018; Karlocai et al., 2021). The (M)unc-13 family has homologous in *Caenorhabditis elegans*, *Drosophila melanogaster* and mammals and is essential for the regulation of neurotransmitter release (Maruyama and Brenner, 1991; Brose et al., 1995; Aravamudan et al., 1999; Augustin et al., 1999). Multiple *C. elegans* studies deciphered protein-protein interactions of Unc-13 during vesicle exocytosis, e.g., between the C-terminal MUN-domain and the SNARE-complex protein Syntaxin (Richmond et al., 2001; Madison et al., 2005; Hammarlund et al., 2007; Guan et al., 2008; Liu et al., 2021). Due to its central role in SNARE-complex formation (M)unc-13 is a key molecular marker of release sites in vertebrates and invertebrates (Böhme et al., 2016; Reddy-Alla et al., 2017; Sakamoto et al., 2018; Dittman, 2019). Furthermore, the N-terminus of Unc-13A in *Drosophila* is essential for presynaptic homeostatic potentiation (PHP, Böhme et al., 2019). Two Unc-13 isoforms in *Drosophila*, Unc-13A and Unc-13B, play different roles in release coupling and their localization at presynaptic AZs was studied using no longer available, subtype-specific antibodies (Piao and Sigrist, 2022). We decided to study the nano-topology of all Unc-13 isoforms at the *Drosophila* neuromuscular junction (NMJ) to analyze the general expression pattern of this important release site marker.

In *Drosophila melanogaster* various tools emerged during the last decade speeding up the generation of genetically marked constructs. Among them is the *Minos*-mediated integration cassette (MiMIC) collection (Venken et al., 2011; Nagarkar-Jaiswal et al., 2015a,b) and the clustered regularly interspaced short palindromic repeats (CRISPR)-CRISPR-associated protein 9 (Cas9) system (Bassett et al., 2013; Gratz et al., 2013, 2014; Kondo and Ueda, 2013; Yu et al., 2013; Port et al., 2014). In the MiMIC collection the MiMIC transposon was randomly inserted into the fly genome creating a catalog of over 7,000 insertions that facilitated the generation of hundreds of green fluorescent protein (GFP)-tagged constructs (Venken et al., 2011; Nagarkar-Jaiswal et al., 2015a).

We found a promising MiMIC insertion within a coding exon of the fly *unc-13* gene allowing endogenous insertion of a genetic reporter into all predicted variants. We inserted an EGFP-FlAsH-StrepII-TEV-3xFlag (GFSTF) tag into the fly genome using a previously described injection method (Venken et al., 2011). Using electrophysiology, we observed undisturbed neurotransmission and PHP expression in these flies. Furthermore, structured illumination microscopy (SIM) revealed undisturbed Unc-13 trafficking to presynaptic AZs. Finally, combining *direct* stochastic optical reconstruction microscopy (*d*STORM, Heilemann et al., 2008; van de Linde et al., 2011) and hierarchical density-based spatial clustering of applications with noise (HDBSCAN, Campello et al., 2013; Mrestani et al., 2021) we uncover Unc-13^GFSTF^ subclusters with ∼26 nm diameter at presynaptic AZs that move toward AZ centers during PHP without enhancement of Unc-13^GFSTF^ protein levels, consistent with earlier described compaction of other AZ components (Mrestani et al., 2021).

## Materials and methods

### Fly stocks

Flies were raised on standard cornmeal and molasses medium at 25^°^C. *Drosophila melanogaster* male 3rd instar larvae of the following strains were used for experiments:

Wildtype: *w*^1118^ [Bloomington *Drosophila* Stock Center (BDSC), Bloomington, IN, United States]. *unc-13*^GFSTF^**: y[1] w[*]; Mi{PT-GFSTF.0}unc-13[MI00468-GFSTF.0] (stock sent out to BDSC). Original MiMIC-strain (MI00468): y[1] w[*]; Mi{y[+ *mDint2]* = *MIC}unc-13[MI00468]/In(4)ci[D], ci[D] pan[ciD]* (BDSC #31015).

### Transgene construction

The Minos-mediated integration cassette (MiMIC) strain MI00468 was used to generate the GFP-tagged Unc-13 line (Venken et al., 2011; Nagarkar-Jaiswal et al., 2015a). Microinjection of the plasmid *pBS-KS-attB1-2-PT-SA-SD-0-EGFP-FlAsH-StrepII-TEV-3xFlag* (*Drosophila* Genomics Resource Center #1298, Venken et al., 2011) and all transgenesis steps were performed at Bestgene Inc. (Chino Hills, CA, United States). Larvae for injection were obtained from crosses of MI00468 males to ΦC31 integrase expressing female virgins. Removal of the genomic ΦC31 integrase source and PCR confirmation of the *attP* sites in MI00468 and of the correct orientation of the recombinase-mediated cassette exchang (RMCE) event were performed by the company. y[1] w[*]; Mi{PT-GFSTF.0}unc-13[MI00468-GFSTF.0] (*unc-13*^GFSTF^**) was shipped as an unbalanced, homozygous viable stock. Molecular confirmation of precise incorporation of the EGFP-FlAsH-StrepII-TEV-3xFlag (GFSTF, sfGFP used in recent versions of the multi-tag, Venken et al., 2011) tag was performed by genomic PCR with primer pairs *fg_30f* (AATGATAAAGGACAGGGACAAGGT) + *fg_31r* (CTGCTTCATGTGATCGGGGT), *fg50_f* (TGGATGGCGA CGTGAAC) + *fg51_r* (GGTTCCATGCAGCATCC) and *fg_58f* (CACAACGTGTACATCACCGC) + *fg_59r* (CTTGA GAACCTGCCGTCCAT). The PCR products were sequenced with *fg_31r*, *fg50_f*, and *fg_58f*.

### Fixation, staining and immunofluorescence

For immunofluorescence imaging of larval neuromuscular junctions and ventral nerve cords, larvae were dissected in ice-cold hemolymph-like solution (HL-3, Stewart et al., 1994), fixed with 4% paraformaldehyde (PFA) in phosphate buffered saline (PBS) for 10 min and blocked for 30 min with PBT (i.e., phosphate buffered saline including 0.05% Triton X-100, Sigma, St. Louis, MI, United States) including 5% natural goat serum (NGS, Dianova, Hamburg, Germany). Primary antibodies were added for overnight staining at 4^°^C. After two short and three long washing steps with PBT (60 min each for data shown in Figure 2C, 20 min each for all other imaging data), preparations were incubated with secondary antibodies for 3 h at room temperature, followed by two short and three 20 min long washing steps with PBT. Preparations were kept in PBS at 4^°^C until imaging. All data were obtained from NMJs formed on abdominal muscles 6/7 in segments A2 and A3. Directly compared data were obtained from larvae stained in the same vial and measured in one imaging session. Primary antibodies were used in the following concentrations: mouse α-Brp (Brp^*Nc*82^, 1:100; AB_2314866, Developmental Studies Hybridoma Bank, Iowa City, IA, United States), rabbit-α-GFP (1:1,000 for *d*STORM or 1:3,000 for Zeiss Axiovert and structured illumination microscopy; A11122, ThermoFisher, Waltham, MA, United States), mouse α-GluRIIA (1:100; AB_528269, Developmental Studies Hybridoma Bank, Iowa City, IA, United States), and Cy5-conjugated α-horseradish-peroxidase (α-HRP, 1:250, AB_2338714, Jackson ImmunoResearch, West Grove, IA, United States).

**FIGURE 1:**
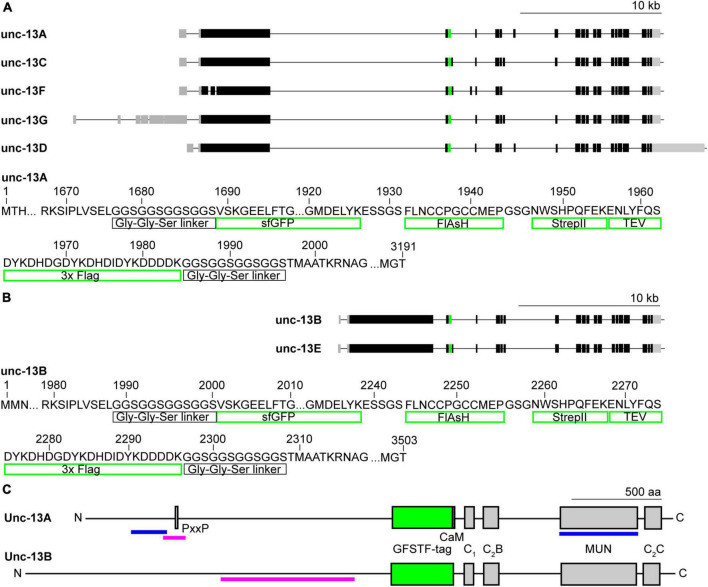
Endogenous tagging of *unc-13* in *Drosophila melanogaster*. **(A,B)** Genetic map of *unc-13* in *Drosophila* with the five annotated transcripts A, C, F, G, and D **(A)** and the two annotated transcripts B and E **(B)**. Black boxes indicate exons, gray boxes introns and green boxes mark the insertion site of the EGFP-FlAsH-StrepII-TEV-3xFlag (GFSTF)-tag located in a coding intron. Lower panels show amino acids of the Unc-13A and Unc-13B fly protein illustrating the specific elements the GFSTF-tag consists of. **(C)** Schematic illustration of Unc-13A and Unc-13B domains: CaM- (Calmodulin), C_1_-, C_2_ B-, C_2_ C-, and MUN-domain and the PxxP-motif. Green box marks GFSTF-tag, magenta bars indicate epitopes of Unc-13A and Unc-13B antibodies (Böhme et al., 2016) and blue bars of N- and C-term antibodies, respectively (Reddy-Alla et al., 2017).

**FIGURE 2:**
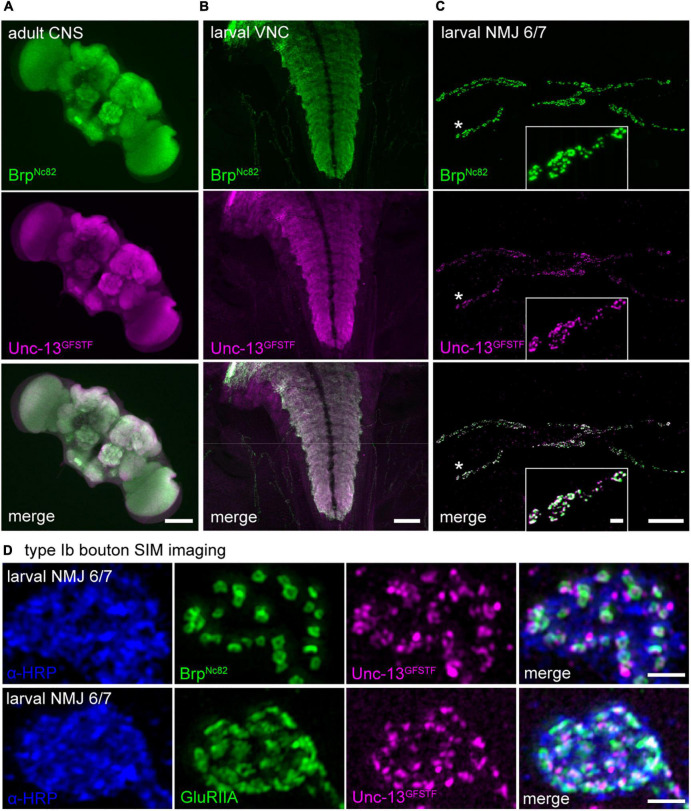
Unc-13^GFSTF^ is expressed in the adult and larval central nervous system and at larval neuromuscular junctions (NMJs). **(A)** Adult central nervous system (CNS) stained with Brp^Nc82^ (green) and rabbit α -green fluorescent protein (α-GFP) antibody to visualize Unc-13^GFSTF^ (magenta). **(B)** Larval ventral nerve cord (VNC) of a male 3rd instar *Drosophila* larva stained with the same antibodies as in **(A)**. **(C)** Co-expression of Brp^Nc82^ (green) and Unc-13^GFSTF^ (magenta) at a 3rd instar larval NMJ on abdominal muscles 6/7 in segment A3. **(D)** Structured illumination microscopy imaging of a type Ib bouton of a larval NMJ on abdominal muscles 6/7 shows pre- and postsynaptic co-localization of α-horseradish peroxidase (α-HRP) (blue), Unc-13^GFSTF^ (magenta), and Brp^Nc82^ (green, upper panel) or GluRIIA receptor subunits (green, lower panel). Scale bars in **(A)** and **(B)** 100 μm, in **(C)** 20 and 2 μm (inset) and in **(D)** 1 μm.

For immunofluorescence imaging of adult brains, fly heads were dissected and brains removed in ice-cold PBS, fixed in 4% PFA in PBS for 30 min and blocked for 30 min with 0.6% PBT including 5% NGS. Incubation in primary antibodies, washing in 0.6% PBT, incubation in secondary antibodies and again washing followed the protocol explained in detail above. The same primary antibodies were used in the following concentrations: mouse α-Brp (Brp^*Nc*82^, 1:100) and rabbit-α-GFP (1:1,000).

### Axiovert imaging and structured illumination microscopy

Preparation, fixation, and staining was performed as described above. Secondary antibodies were used in the following concentrations for larval neuromuscular junctions, ventral nerve cords and adult fly brains: goat α-rabbit conjugated Cy3 (1:500, AB_2338006, Jackson ImmunoResearch, West Grove, IA, United States) and goat α-mouse conjugated Alexa Fluor488 (1:500, A32723, Invitrogen, Waltham, MA, United States). Larval preparations were mounted in Vectashield (Vector Laboratories, Burlingame, CA, United States) for Axiovert imaging or Prolong Glass (ThermoFisher, Waltham, MA, United States) for structured illumination microscopy. Images were acquired at room temperature from NMJs on muscles 6/7 in segments A2 and A3. An Apotome System (Zeiss, Jena, Germany, Axiovert 200M Zeiss, objective 63x, NA 1.4, oil) was used for low-resolution characterization of larval NMJs, larval ventral nerve cords and adult brains (Figures 2A–C). For SIM we used a Zeiss Elyra S.1 structured illumination microscope equipped with a sCMOS camera (pco.edge 5.5 m) and an oil-immersion objective (Plan-Apochromat 63x, 1.4 NA). Lasers with 488, 531, and 641 nm wavelength were used. Z step size was set to 0.1 μm, and imaging was performed using five rotations of the grating at five different phase steps. Fourier transformation of structured illumination images was performed using ZEN software (ZEISS Efficient Navigation, Carl Zeiss, Jena, Germany), and subsequent analysis was done with ImageJ.

### *Direct* stochastic optical reconstruction microscopy

*direct* stochastic optical reconstruction microscopy imaging of the specimen was performed essentially as previously reported (Ehmann et al., 2014; Paul et al., 2015, 2022; Mrestani et al., 2021). Preparations were incubated with respective primary antibodies as described above. The following secondary antibodies were used: goat α-rabbit F(ab’)_2_ fragments labeled with Alexa Fluor647 (1:500; A21246, Thermofisher, Waltham, MA, United States) and goat α-mouse IgGs labeled with Alexa Fluor532 (1:500; A11002, ThermoFisher, Waltham, MA, United States). After staining, larval preparations were incubated in 100 mM mercaptoethylamine (MEA, Sigma, St. Louis, MI, United States) in a 0.2 M sodium phosphate buffer, pH ∼7.9, to allow reversible switching of single fluorophores during data acquisition (van de Linde et al., 2008). Images were acquired using an inverted microscope (Olympus, Tokio, Japan, IX-71, 60x, NA 1.49, oil immersion) equipped with a nosepiece-stage (IX2-NPS, Olympus, Tokio, Japan). 647 nm (F-04306-113, MBP Communications Inc., Quebec, Kanada) and 532 nm (gem 532, Laser Quantum, Stockport, United Kingdom) lasers were used for excitation of Alexa Fluor647 and Alexa Fluor532, respectively. Laser beams were passed through clean-up filters (BrightLine HC 642/10 and Semrock, ZET 532/10, respectively), combined by two dichroic mirrors (LaserMUX BS 514-543 and LaserMUX BS 473-491R, 1064R, F38-M03, AHF Analysentechnik, Tübingen, Germany), and directed onto the probe by an excitation dichroic mirror (HC Quadband BS R405/488/532/635, F73-832, AHF Analysentechnik, Tübingen, Germany). The emitted fluorescence was filtered with a quadband-filter (HC-quadband 446/523/600/677, Semrock, West Henrietta, NY, United States) and a long pass- (Edge Basic 635, Semrock, West Henrietta, NY, United States) or bandpass-filter (Brightline HC 582/75, Semrock, West Henrietta, NY, United States) for the red and green channel, respectively, and divided onto two cameras (iXon Ultra DU-897-U, Andor, Oxford, United Kingdom) using a dichroic mirror (HC-BS 640 imaging, Semrock, West Henrietta, NY, United States). For the red channel, image resolution was 127 × 127 nm per pixel to obtain super-resolution of Unc-13^GFSTF^. For the green channel, image resolution was 130 x 130 nm per pixel. Single fluorophores were localized and high resolution-images were reconstructed with rapi*d*STORM (Heilemann et al., 2008; Wolter et al., 2010, 2012; van de Linde et al., 2011)^1^. Only fluorescence spots with an A/D count over 12,000 were analyzed and a subpixel binning of 10 nm px^–1^ was applied.

### Analysis of localization data

Localization data were analyzed essentially as described previously (Mrestani et al., 2021) with an extension for two-channel localization data. Analysis was performed with custom written Python code (language version 3.6)^2^ and the web-based Python interface Jupyter^3^. Localization tables from rapi*d*STORM were directly loaded and analyzed. Prior to the Python-based analysis the regions of interest (ROI) were masked in the reconstructed, binned images from rapi*d*STORM using FIJI (Schindelin et al., 2012). These ROIs corresponded to the terminal six boutons. For cluster analysis we used the Python implementation of HDBSCAN (McInnes et al., 2017)^4^ which takes “minimum cluster size” and “minimum samples” as the main free parameters. In the first step we extracted Brp clusters in the Alexa Fluor532 channel, corresponding to individual AZs, with the combination 100 and 25 for minimum cluster size and minimum samples (Figure 4Aiii; compare Mrestani et al., 2021). All unclustered localizations were discarded from further analysis. Brp clusters were used to remove noise from the Unc-13^GFSTF^ Alexa Fluor647 channel such that all Unc-13^GFSTF^ localizations with an euclidean distance >20 nm to Brp localizations were discarded. The H function (Figure 4B) as derivative of Ripley’s K function was computed using Python package Astropy (Robitaille et al., 2013) according to our previous algorithm (Mrestani et al., 2021) for the denoised Unc-13^GFSTF^ localizations and for the random Poisson distribution. Curves for display were averaged (mean ± SD). The function was evaluated in nm steps for radii from 0 to 120 nm and without correction for edge effects. A second HDBSCAN to extract the individual Unc-13^GFSTF^ subclusters (SC) was performed with minimum cluster size seven and minimum samples two to get SCs with a radius that matches the maximum of the H function. Unc-13^GFSTF^ SCs were assigned to individual Brp clusters by computing the euclidian distance between the center of mass (c.o.m.) of each SC and the c.o.m. of each Brp cluster and selecting the lowest distance. In that way, the number of SCs per AZ could be quantified. The distance between the c.o.m.s is referred to as radial distance and was computed for each individual AZ as mean of the assigned Unc-13^GFSTF^ SCs. To quantify cluster areas, we computed 2D alpha shapes using CGAL (Computational Geometry Algorithms Library)^5^ in Python. To get the alpha shapes of Brp clusters and Unc-13^GFSTF^ SCs we choose α-values of 800 and 300 nm^2^, respectively. Exclusion criteria for Brp clusters were area <0.03 and >0.3 μm^2^ (Mrestani et al., 2021). Unc-13^GFSTF^ SCs that were assigned to those Brp clusters were also excluded from further analysis, as well as SCs where alpha shape determination failed due to sparse signal that yielded SC areas of 0 μm^2^. The Unc-13 area per AZ (Figure 4E) was computed as the sum of all Unc-13 SC areas belonging to an individual AZ. Brp cluster circularity was computed as described previously (Mrestani et al., 2021). For the analysis of Unc-13^GFSTF^ superclusters (SpC, Figures 5B,D,E and Supplementary Figures 2B,C) and distances between SC c.o.m.s (Figure 5C and Supplementary Figure 2A) only AZs with a circularity ≥0.6 were selected. The Python module scikit-learn (Pedregosa et al., 2011) was used to compute distances between SC c.o.m.s. To extract SpCs, HDBSCAN was performed taking the SC c.o.m.s of an individual AZ as input (minimum cluster size and minimum samples two, respectively). The cluster selection method was changed to “leaf” clustering, which comprises a tendency to more homogeneous clusters by extracting those that lie on leaf nodes of the cluster tree rather than the most stable clusters. The default setting “excess of mass,” that was used for all other cluster analyses throughout this study, delivered similar statistical results and median values (data not shown) but less intuitive clustering. For the statistical comparison of SpC numbers per AZ between experimental groups (Figure 5D) only AZs where SpCs could be detected were included. The SpC c.o.m. was defined as the c.o.m. of its respective SC c.o.m.s and the euclidean distance between these SC c.o.m.s and the SpC c.o.m. was computed as mean per SpC.

**FIGURE 3:**
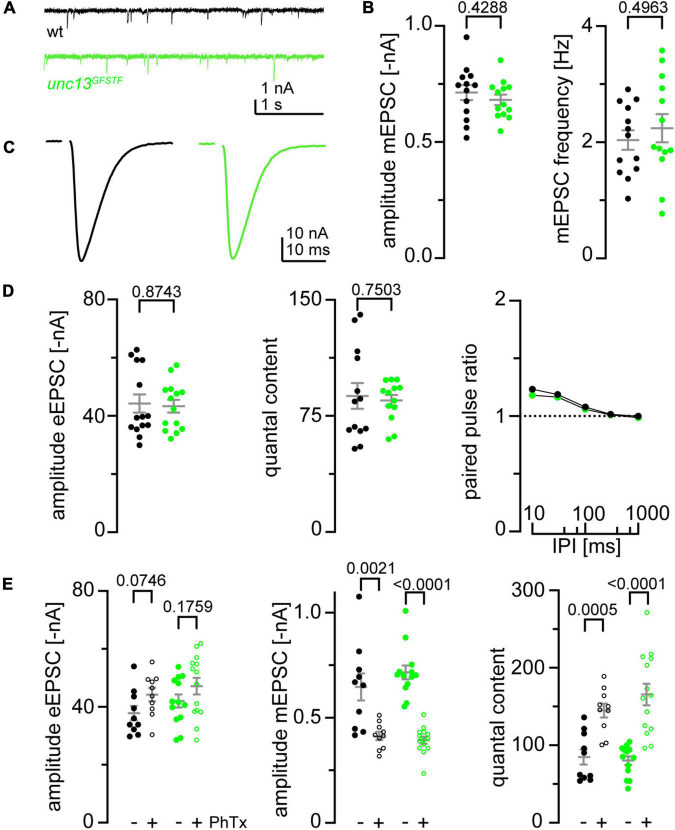
Endogenous tagging of *unc-13* leaves synaptic function and presynaptic homeostatic potentiation undisturbed. **(A)** Representative traces of miniature excitatory postsynaptic currents (EPSCs) recorded in 1 mM Ca^2+^ at wildtype (wt, black) and *unc-13*^GFSTF^** (green) neuromuscular junctions (NMJs). **(B)** miniature excitatory postsynaptic currents (mEPSC) amplitude and frequency are unaltered in *unc-13*^GFSTF^** larvae (green, *n* = 13 NMJs from seven larvae) compared to wt (black, *n* = 13 NMJs from eight larvae). **(C)** Representative traces of evoked EPSCs recorded in 1 mM extracellular Ca^2+^ in both genotypes. **(D)** Evoked excitatory postsynaptic currents (eEPSC) amplitude, quantal content, and paired-pulse-ratios measured with different interpulse intervals (IPI; 10, 30, 100, 300, and 1000 ms) remain unaltered in *unc-13*^GFSTF^** animals (wt: *n* = 14 NMJs from eight larvae; *unc-13*^GFSTF^**: *n* = 14 NMJs from seven larvae). **(E)** eEPSC amplitude, mEPSC amplitude and quantal content in wt (black) and *unc-13*^GFSTF^** (green) animals treated with Philanthotoxin (PhTx) in dissolved in dimethyl sulfoxide (dmso) (+, open circles) or dmso (–, filled circles). *unc-13*^GFSTF^** larvae still exhibit presynaptic homeostatic potentiation in response to PhTx stimulation (wt: 10 NMJs from seven larvae in dmso, 10 NMJs from five larvae in PhTx; *unc-13*^GFSTF^**: 13 NMJs from six larvae in dmso, 14 NMJs from seven larvae in PhTx). Whisker plots represent mean ± SEM, scatter plots show individual data points, individual *p*-values are indicated.

**FIGURE 4:**
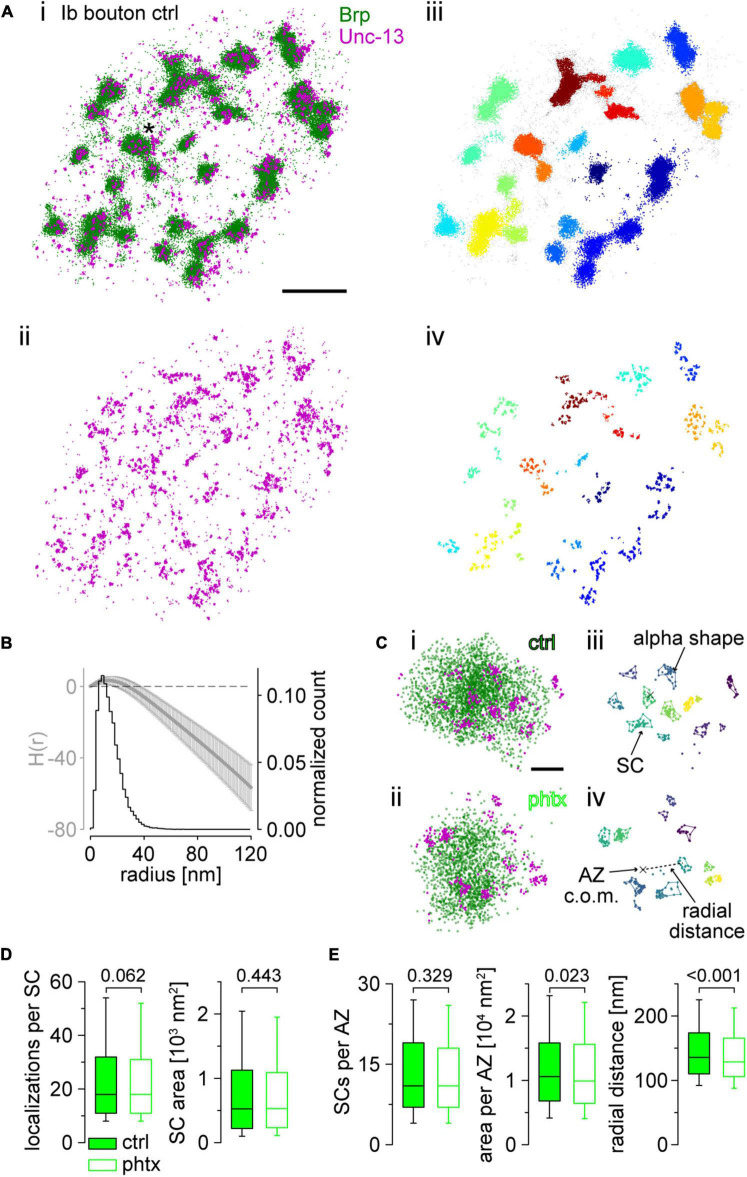
Super-resolution imaging of Unc-13^GFSTF^ at presynaptic active zones (AZs) reveals nanoscale reorganization in presynaptic homeostatic potentiation (PHP). **(A)** Scatter plots of two-channel *direct* stochastic optical reconstruction microscopy (*d*STORM) localizations of an unc-13^GFSTF^ type Ib bouton from a control (ctrl) animal co-stained with α-green fluorescent protein (α-GFP) antibody labelled with Alexa Fluor647 conjugated F(ab’)_2_ fragments for visualization of Unc-13^GFSTF^ (magenta) and Brp^Nc82^ labelled with Alexa Fluor532 conjugated IgGs (green). **(Ai)** Overlay of the two channels. Asterisk marks the enlarged region in **(Ci)**. **(Aii)** Red channel showing Unc-13GFSTF localizations of the same bouton as in **(Ai)**. **(Aiii)** Brp localizations from **(Ai)** with clusters extracted by HDBSCAN and individual AZs in different colors. Unclustered localizations are shown in gray. **(Aiv)** Unc-13GFSTF localizations from **(Aii)** with all localizations with euclidean distance >20 nm to Brp localizations removed. The removed signal is considered noise. Individual Unc-13^GFSTF^ subclusters (SCs) were extracted by HDBSCAN and assigned to nearest AZs by color. **(B)** Averaged H function (black, mean ± SD) from *n* = 2,040 Unc-13^GFSTF^ first level clusters obtained from 22 NMJs from nine animals (maximum of the curve indicates a mean sc. radius of 13 nm) and histogram (gray) of the mean radius from *n* = 20,037 Unc-13^GFSTF^ SCs [estimated from SC area under the assumption of a circular area, median (25th–75th percentile): 12.9 (8.4–18.9) nm]. Dashed black line indicates the prediction for a random Poisson distribution. **(C)** Scatter plots of Ib AZs from a ctrl **(Ci,iii)** and a Philanthotoxin treated animal [phtx, (**Cii,iv)**]. **(Ci,ii)** Original scatter plots Unc-13^GFSTF^ and Brp^Nc82^ *d*STORM localizations. Unclustered localizations are not shown in both channels. **(Ciii,iv)** Unc-13^GFSTF^ subclusters (SCs) extracted by HDBSCAN, that are assigned to an individual ctrl **(Ciii)** and phtx **(Civ)** AZ, in different colors. Colored lines indicate alpha shapes used for area determination. Centers of mass (c.o.m.) of the corresponding AZ (cross) are indicated. Dashed line shows the euclidian distance between the AZ c.o.m and an SC c.o.m., referred to as radial distance. **(D)** Number of localizations per Unc-13^GFSTF^ SC and SC area in ctrl [filled boxes, *n* = 20,037 SCs from 22 neuromuscular junctions (NMJs) and nine animals] and phtx (open boxes, *n* = 20,393 SCs from 23 NMJs and nine animals) shown as box plots (horizontal lines show median, box boundaries 25th and 75th percentiles, whiskers 10th and 90th percentiles). **(E)** Number of Unc-13^GFSTF^ SCs, total Unc-13^GFSTF^ area and radial distance of Unc-13^GFSTF^ SCs per AZ in ctrl (*n* = 1,462 AZs), and phtx (*n* = 1,521 AZs). Scale bars in **(A)** 1 μm, in **(C)** 100 nm.

**FIGURE 5:**
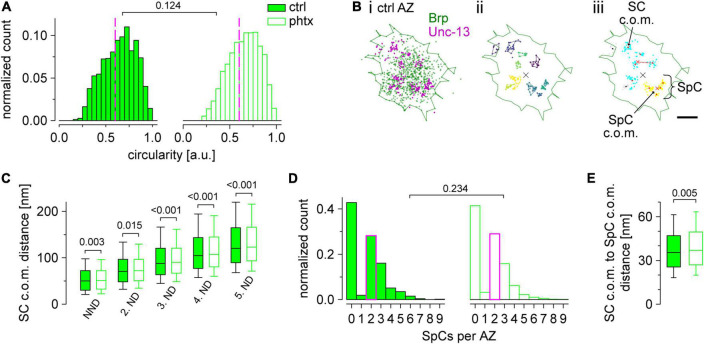
Unc-13^GFSTF^ superclusters are enlarged in presynaptic homeostatic potentiation (PHP). **(A)** Active zone (AZ) circularity computed for Brp clusters in *unc-13*^GFSTF^** after dissolved in dimethyl sulfoxide (dmso) [control, ctrl; filled bars, *n* = 1,462 AZs from 22 neuromuscular junctions (NMJs) and nine animals] or philanthotoxin (PhTx) treatment (open bars, *n* = 1,521 AZs from 23 NMJs and nine animals). Dashed magenta lines indicate the cutoff circularity of ≥0.6 a.u. used for further analyses. **(B)** Scatter plots of a representative, circular AZ from a ctrl type Ib bouton. Green line indicates the alpha shape used for area determination of the AZ. **(Bi)** Original scatter plots of Unc-13^GFSTF^ and Brp^Nc82^ *direct* stochastic optical reconstruction microscopy (*d*STORM) localizations. **(Bii)** Unc-13^GFSTF^ subclusters (SCs) extracted by hierarchical density-based spatial clustering of applications with noise (HDBSCAN) in different colors. Unclustered localizations are not shown. Colored lines indicate distinct alpha shapes. The center of mass (c.o.m.) of the corresponding AZ (black cross) is shown. **(Biii)** Two individual superclusters (SpCs), indicated by blue and yellow colors, extracted by HDBSCAN from the c.o.m.s (red dots) of Unc-13^GFSTF^ SCs shown in (Bii). Black dots represent SC c.o.m.s that are unclustered according to HDBSCAN analysis and gray dots show localizations of the corresponding SCs. Red crosses indicate c.o.m.s of SpCs. Dashed red line indicates the distance between a SC c.o.m. and its respective SpC c.o.m. **(C)** Nearest neighbor distance (NND) and 2nd to 5th neighbor distance (ND) of Unc-13^GFSTF^ SCs at circular AZs for ctrl (*n* = 10,461, 10,425, 10,329, 10,185, and 9,945 distances from 876, 858, 826, 790, and 742 AZs for NND and 2nd to 5th ND, respectively) and phtx (*n* = 10,988, 10,940, 10,856, 10,688, and 10,493 distances from 927, 903, 875, 833, and 794 AZs for NND and 2nd to 5th ND, respectively). **(D)** Number of SpCs per AZ. Median values, indicated in magenta, as well as statistical comparison refers to AZs with at least one SpC (ctrl: *n* = 501 from 22 NMJs and nine animals; phtx: *n* = 543 AZs from 23 NMJs and nine animals). **(E)** Distance between c.o.m.s of SCs and SpCs for both groups at AZs with at least one SpC (*n* = 1,417 and 1,497 SpCs for ctrl and phtx, respectively).

### Electrophysiology

Two-electrode voltage clamp recordings (Axoclamp 2B amplifier, Digidata 1440A; Molecular Devices, San José, CA, United States) were obtained from abdominal muscle 6 in segments A2 and A3 as previously described (Paul et al., 2022). All measurements were obtained at room temperature in HL-3 (Stewart et al., 1994) with the following composition (in mM): NaCl 70, KCl 5, MgCl_2_ 20, NaHCO_3_ 10, trehalose 5, sucrose 115, Hepes 5, and CaCl_2_ 1, pH adjusted to 7.2. Intracellular electrodes had resistances of 10–30 MΩ and were filled with 3 M KCl. For analysis, only cells with an initial membrane potential of at least –50 mV and a membrane resistance of ≥4 MΩ were included. During recordings, cells were clamped at a holding potential of –80 mV (minis) or –60 mV (evoked EPSCs). To evoke synaptic currents, nerves were stimulated *via* a suction electrode with pulses of 300 μs length and typically at 12 V (Grass S48 stimulator and isolation unit SIU5; Astro-Med, West Warwick, United States). Signals were low-pass filtered at 10 kHz and analyzed in Clampfit 11.1 (Molecular Devices, San José, CA, United States). Paired-pulse recordings were performed with interstimulus intervals of (in ms: 10, 30, 100, 300, and 1,000). Between recordings, cells were given a 10 s rest. For analysis, 5–10 traces per interval were averaged. The amplitude of the second response in 10 ms interpulse recordings was measured from the peak to the point of interception with the extrapolated first evoked excitatory postsynaptic current (eEPSC) as described previously (Hallermann et al., 2010; Weyhersmüller et al., 2011). To assess basal synaptic transmission 10 EPSCs evoked at 0.2 Hz were averaged per cell. The quantal content was estimated by dividing the mean eEPSC amplitude by the mean evoked excitatory postsynaptic current (mEPSC) amplitude measured in the same cell. mEPSC amplitudes were corrected for the more hyperpolarized holding potential (Hallermann et al., 2010).

### Philanthotoxin treatment

Philanthotoxin 433 tris (trifluoroacetate) salt (PhTx, CAS 276684-27-6, Santa Cruz Biotechnology, United States) was dissolved in dimethyl sulfoxide (DMSO) to obtain a stock solution of 4 mM and stored at –20^°^C. For each experiment, the respective volume was further diluted with freshly prepared HL-3 to a final PhTx concentration of 20 μM in 0.5% DMSO. Control experiments were performed with the same DMSO concentration in HL-3. PhTx treatment of semi-intact preparations was performed essentially as described previously (Frank et al., 2006; Mrestani et al., 2021). In brief, larvae were pinned down in calcium-free, ice-cold HL-3 at the anterior and posterior endings, followed by a dorsal incision along the longitudinal axis. Larvae were incubated in 10 μl of 20 μM PhTx in DMSO for 10 min at room temperature. Following this incubation time, PhTx was replaced by HL-3 and larval preparations were completed, followed by electrophysiological measurements or *d*STORM imaging.

### Statistics

Statistical analyses were performed with Sigma Plot 13 (Systat Software, Düsseldorf, Germany) or GraphPad Prism 9 (San Diego, United States). Shapiro-Wilk was used to test normality. If data were not normally distributed, we used the non-parametric Mann-Whitney rank sum test for statistical analysis and reported data as median (25th–75th percentile). If data were normally distributed, they were reported as mean ± SD unless indicated otherwise. In box plots, horizontal lines represent median, boxes quartiles and whiskers 10th and 90th percentiles. Scatter plots show individual data points unless indicated otherwise. Bin counts in histograms were normalized to the total number of observed events which was set to one. All plots were produced with Sigma Plot. Figures were assembled using Adobe Illustrator (Adobe, 2015.1.1 release, San José, CA, United States). Supplementary Tables 1–4 contain all numerical values not stated in text and figure legends including *p*-values and samples sizes.

### Code and data availability

The authors declare that custom written Python code and all data sets supporting the findings of this work are available from the corresponding authors.

## Results

### Endogenous tagging of unc-13

To generate an endogenously tagged *unc-13* locus, we screened the previously established MiMIC library (Venken et al., 2011; Nagarkar-Jaiswal et al., 2015a,b) for an appropriate line. The MiMIC transposon harbors selection markers (*yellow*^+^ and enhanced GFP [EGFP]) and two inverted *attP* sites for recombinase-mediated cassette exchange (RMCE), as well as a gene-trap cassette (Venken et al., 2011). The insertion MI00468 carries a MiMIC element in a coding intron and, *via* RMCE with a protein-trap plasmid, allows the generation of a new exon that gets spliced in all annotated *unc-13* transcripts (Figures 1A,B). This strategy was applied to insert a multi-tag, consisting of superfolder (sf) GFP (sfGFP), a modified Fluorescein arsenical helix binder (FlAsH) binding tetracysteine motif, the StrepII peptide tag, a TEV protease cleavage site and the Flag peptide tag [EGFP-FlAsH-StrepII-TEV-3xFlag (GFSTF) tag], using a previously published protein-trap plasmid (see Materials and methods; Venken et al., 2011; Nagarkar-Jaiswal et al., 2015b). The multi-tag combines the advantages of different protein and peptide tags including several possibilities for antibody staining, live imaging and RNAi knockdown. GFSTF is inserted at the same amino acid positions in the two major isoforms Unc-13A (Figure 1A) and Unc-13B (Figure 1B) but is C-terminally followed by alternative amino acids in Unc-13C, E, and F, resulting from the additional exon in the respective transcripts. The multi-tag is located at the C-terminal side of the large unstructured N-Terminus and is followed by all annotated domains of the proteins (Figure 1C). The animals are homozygous viable and appear healthy (e.g., emerging and effective flight). In the following, we focus on the detection of sfGFP using a combination of primary IgG antibodies and secondary F(ab’)_2_ fragments to establish a protocol comparable to earlier applications of *d*STORM imaging at the *Drosophila* NMJ (Ehmann et al., 2014; Paul et al., 2015, 2022; Mrestani et al., 2021). We refer to the fusion protein and the genotype as Unc-13^GFSTF^ and *unc-13*^GFSTF^**, respectively.

### Expression of Unc-13^GFSTF^ in the adult and developing *Drosophila* nervous system

To assess expression of the endogenously tagged Unc-13 in the *Drosophila* nervous system, we performed immunostaining using a polyclonal antibody against GFP (see Materials and methods) for detection of the sfGFP within the GFSTF-tag and a well-characterized, highly specific monoclonal antibody Brp^*Nc*82^ mapping to the C-terminal region of Bruchpilot (Brp, Kittel et al., 2006; Fouquet et al., 2009; Figure 2). We found strong and specific expression of Unc-13^GFSTF^ in the adult and larval central nervous system (Figures 2A,B). In addition, we detected reliable Unc-13^GFSTF^ expression at larval NMJs in co-expression with Brp (Figure 2C). Since Brp is an abundant presynaptic epitope and covers the spatial extent of an individual presynaptic AZ (Mrestani et al., 2021) we set out to analyze the co-localization of Brp and Unc-13^GFSTF^. We employed SIM at individual type Ib boutons that show structural adaptation during presynaptic homeostasis (Weyhersmüller et al., 2011). Our data revealed co-localization of Unc-13^GFSTF^ and Brp at presynaptic AZs and of Unc-13^GFSTF^ and postsynaptic glutamate receptors using a monoclonal antibody against the GluRIIA subunit (Figure 2D). We conclude that incorporation of the fluorescent GFSTF-tag at the endogenous locus of *unc-13* in *Drosophila* reports reliable and reasonable localization of the protein at presynaptic AZs allowing further investigations at the nanoscale level (compare Figures 4, 5).

### Normal synaptic function of *unc-13*^GFSTF^** neuromuscular junctions

To evaluate spontaneous and evoked synaptic release at larval NMJs of *unc-13*^GFSTF^** we performed two-electrode voltage clamp recordings (TEVC, Figure 3). First, mEPSCs were recorded to examine spontaneous vesicle release (Figure 3A). mEPSC amplitudes and the frequency of spontaneous fusion events were unchanged in *unc-13*^GFSTF^** compared to wildtype controls (Figure 3B). In addition, eEPSCs in response to nerve stimulation were unaltered in *unc-13*^GFSTF^** compared to controls (Figures 3C,D). Furthermore, quantal content was unchanged in *unc-13*^GFSTF^** (Figure 3D). Next, we tested whether insertion of the tag alters synaptic short-term plasticity. We found paired pulse ratios (PPR) unchanged in *unc-13*^GFSTF^** (Figure 3D). Taken together, these data reveal no differences between *unc-13*^GFSTF^** and control larvae in basal transmission properties of spontaneous and evoked synaptic release, making the endogenously tagged Unc-13 variant a valuable tool for the assessment of AZ function and structure.

### Presynaptic homeostatic potentiation in *unc-13*^GFSTF^**

In addition to its role in docking and priming of synaptic vesicles Unc-13 is involved in diverse presynaptic plasticity processes including Ca^2+^, DAG, or RIM-dependent short-term plasticity (Dulubova et al., 2005; Shin et al., 2010; Xu et al., 2017) and presynaptic homeostatic potentiation (PHP, Böhme et al., 2019). In *Drosophila*, upregulated Unc-13A levels and increased numbers of Unc-13A nanomodules at presynaptic AZs in acute and chronic PHP were observed (Böhme et al., 2019). Furthermore, functional PHP is abolished in *unc-13A* null mutants and the functional dependence can be attributed to the Unc-13A N-terminus. We decided to probe if the GFSTF-tagged *unc-13 Drosophila* strain is suitable for PHP analyses. To this end we measured the electrophysiological response to an acute homeostatic challenge using Philanthotoxin (PhTx) in *unc-13*^GFSTF^** animals compared to control larvae (Figure 3E). We found that upon PhTx treatment *unc-13*^GFSTF^** larvae showed the same increase in quantal content and thus evoked EPSC restoration as control larvae, thus, indicating that *unc-13*^GFSTF^** animals can still exhibit functional PHP to the full extent. We conclude that incorporation of the GFSTF-tag into the endogenous unc-13 locus does not disrupt this form of presynaptic plasticity *in vivo*.

### Unc-13^GFSTF^ subclusters at the active zone mesoscale are reorganized in presynaptic homeostatic potentiation

Next, we performed localization microscopy in terms of two-color *d*STORM (Figure 4; Heilemann et al., 2008; van de Linde et al., 2011; Löschberger et al., 2012; Ehmann et al., 2014; Paul et al., 2015; 2022; Mrestani et al., 2021; Pauli et al., 2021). We used Brp^*Nc*82^ and a polyclonal antibody against GFP for detection of Unc-13^GFSTF^ in type Ib boutons and found Unc-13^GFSTF^ in discrete co-localization with Brp (Figure 4A). The HDBSCAN analysis extracted individual Unc-13^GFSTF^ subclusters (SCs) matching the maximum of the H function with diameters of ∼26 nm (Figures 4B,C). We aimed to investigate if induction of acute PHP leads to reorganization of Unc-13^GFSTF^ within the presynaptic AZ. Thus, we compared Brp and Unc-13^GFSTF^ localization data in PhTx treated preparations (phtx) and DMSO controls (ctrl, Figures 4C–E and Supplementary Figure 1). Using Alexa Fluor532 for detection of Brp^*Nc*82^ we found decreased Brp cluster areas with similar localization numbers in phtx, as described before using Alexa Fluor647 (Supplementary Figure 1; compare Mrestani et al., 2021). Analysis of Unc-13^GFSTF^ SCs revealed no changes in localization numbers per SC or SC area in phtx (Figure 4D) with equal numbers of SCs per AZ (Figure 4E). Yet, the overall Unc-13^GFSTF^ area per AZ and the radial distance between the AZ c.o.m. and individual Unc-13^GFSTF^ SC c.o.m.s was decreased in phtx (Figure 4E). Our data reveal that Unc-13^GFSTF^ forms distinct subclusters within the AZ in co-localization with Brp which are reorganized during acute PHP.

### *Direct* stochastic optical reconstruction microscopy reveals formation of Unc-13^GFSTF^ superclusters at active zones

Our super-resolution analysis so far showed ∼11 Unc-13^GFSTF^ SCs per AZ (Figure 4E). However, the number of docked vesicles at *Drosophila* type Ib boutons is known to be lower (Böhme et al., 2016; Reddy-Alla et al., 2017). Furthermore, the diameter of individual SCs is only ∼26 nm and thus smaller than the diameter of individual synaptic vesicles (Karunanithi et al., 2002). Therefore, we wondered whether Unc-13^GFSTF^ SCs might be spatially organized at a higher level into release sites. We employed a second round of HDBSCAN analysis focusing only on AZs in planar view (indicated by circularity values ≥0.6, Figure 5A). As illustrated in Figure 5B this evaluation revealed what we termed Unc-13^GFSTF^ superclusters (SpC), each containing several Unc-13^GFSTF^ SCs (Figure 5B). Distances between nearest-neighbors of all Unc-13^GFSTF^ SCs within an AZ increased in phtx compared to ctrl (Figure 5C and Supplementary Figure 2A). However, whereas median, 10th, 25*^th^*, and 75*^th^* percentiles were increased, the 90th percentile was decreased in phtx suggesting narrower distance distributions (Figure 5C). This indicates a more even allocation of Unc-13^GFSTF^ SCs at the available space in PHP. Next, we asked whether Unc-13^GFSTF^ superclustering occurs at all AZs at the NMJ and whether it is influenced by PHP. We found that most AZs contained 2–3 SpCs in both phtx and ctrl, however, a large fraction of AZs did not show superclustering at all (Figure 5D). Overall, about 50% of the Unc-13^GFSTF^ SCs of an individual AZ were clustered and one individual SpC consisted of ∼3 Unc-13^GFSTF^ SCs in both phtx and ctrl (Supplementary Figures 2B,C). Finally, we analyzed the distance between individual Unc-13^GFSTF^ SC c.o.m.s and their corresponding SpC c.o.m. in PHP (Figure 5E). Remarkably, this distance was increased in phtx compared to ctrl indicating expansion of Unc-13^GFSTF^ SpCs following the homeostatic challenge. In summary, PHP induces a more homogenous distribution of Unc-13^GFSTF^ SCs forming enlarged SpCs that may correlate with increased vesicle traffic.

## Discussion

We describe a MiMIC insertion of the EGFP-FlAsH-StrepII-TEV-3xFlag (GFSTF)-tag within a coding exon of the *Drosophila unc-13* gene allowing visualization of all Unc-13 isoforms expressed within the fly nervous system (Figure 1). Using super-resolution imaging we demonstrate that Unc-13^GFSTF^ localizes at *Drosophila* AZs while electrophysiological characterization reveals no disturbances of spontaneous or evoked release and PHP expression (Figures 2, 3). Furthermore, employing this newly generated tool and cluster analysis of two-channel localization data we show distinct Unc-13^GFSTF^ SCs of about 26 nm diameter and ∼520 nm^2^ area containing ∼18 localizations translating into 2–3 Unc-13^GFSTF^ proteins per SC at presynaptic AZs which are compacted during PHP (Figure 4).

In mammals, Munc-13 exists in nano-assemblies of 5–10 protein copies at the presynaptic AZ and is responsible for recruiting Syntaxin-1 to promote vesicle exocytosis (Sakamoto et al., 2018). It has been shown that the number of release sites equals the number of Munc13-1 clusters within the AZ and that every vesicle needs to bind to a Munc13-1 cluster to get released (Sakamoto et al., 2018). A recent study using quantitative total internal reflection fluorescence (TIRF) microscopy and stepwise photobleaching underlined the functional importance of Munc13-1 clustering for vesicle docking and fusion (Li et al., 2021): a minimum of 6 Munc13-1 copies and especially the C-terminal C_2_C domain are necessary for efficient vesicle binding to lipid bilayers, thus, nano-clustering can be considered an inherent property of Munc13-1. However, the size of (M)unc-13 nano-assemblies is still debatable. Sakamoto et al. (2018) measured about 45 nm diameter in primary hippocampal neuronal cultures of 21 days old rats using STORM imaging. Using 2D *d*STORM imaging and the glutamatergic *Drosophila* NMJ in a newly generated GFSTF-tag MiMIC line we here describe individual Unc-13^GFSTF^ SCs of 26 nm diameter (which is well below the size of an individual synaptic vesicle in *Drosophila*, Karunanithi et al., 2002) and ∼520 nm^2^ area (Figure 4). Differences in model organisms and synapses, imaging technique and analysis algorithms are likely to explain the numerical differences. Here, we used an established HDBSCAN analysis algorithm for investigation of two-channel localization data to study two AZ epitopes in close spatial relation. Brp clusters in the Alexa Fluor532 channel served as a “mask” for determination of AZ extent. Remarkably, using a fluorophore with less favorable photo-physics for detection of Brp (Heilemann et al., 2008; van de Linde et al., 2011) we found similar cluster areas compared to previous Alexa Fluor647 measurements (Supplementary Table 4; compare Mrestani et al., 2021; Paul et al., 2022). The two-channel analysis allowed precise determination of the amount and extent of Unc-13^GFSTF^ SCs within the presynaptic AZ, even enabling further detailed analysis after PHP induction (see below). Distinct labeling strategies, experimental organisms and synapses as well as the localization precision of the applied microscopic techniques might explain the different dimensions of Unc-13 clusters in our and previous work. *d*STORM as a variant of localization microscopy in principle may allow to count molecules (Löschberger et al., 2012; Ehmann et al., 2014). In this study we found 11–32 (25th–75th percentile) Unc-13^GFSTF^ localizations per SC which can be translated into a certain number of Unc-13^GFSTF^ molecules. Considering the 8.1 ± 0.2 (mean ± SEM) localizations per Alexa Fluor647-labeled F(ab’)_2_ fragment (Löschberger et al., 2012; Ehmann et al., 2014) we assume that the 306 localizations per AZ (Supplementary Table 4) may correspond to 38 Unc-13^GFSTF^ proteins distributed in 11 SCs, each containing on average 3.5 Unc-13^GFSTF^ molecules. However, we used a polyclonal primary antibody whereas Ehmann et al. (2014) used a monoclonal antibody. Thus, the true number of Unc-13^GFSTF^ molecules per SC might be lower (i.e., one or two molecules).

Additionally, we describe a higher-level organization that occurs in roughly 60% of AZs, in the form of about two Unc-13^GFSTF^ SpCs per AZ, each containing about three SCs (Figure 5 and Supplementary Figure 2). Strikingly, the number of SpCs per AZ equals the previously determined number of docked vesicles (Böhme et al., 2016; Reddy-Alla et al., 2017). One might speculate that the Unc-13^GFSTF^ SpCs reported here are the mesoscale counterpart of a (M)unc-13 ring. The ring and it’s MUN domains are aligned to 18 Synaptotagmin C_2_B domains and were proposed to serve as a platform for SNAREpin assembly and the subsequent fusion of a synaptic vesicle (Rothman et al., 2017). The enlarged Unc-13^GFSTF^ SpC diameter from ∼70 to ∼74 nm (Figure 5E and Supplementary Table 4) could reflect enhanced vesicle traffic accompanied by an increased abundance of AZs captured during the process of vesicle fusion.

Furthermore, the number of Unc-13^GFSTF^ molecules per AZ is likely to depend on the current status of the synapse, as the amounts of other essential AZ components also change in an activity-dependent manner (Kittel et al., 2006; Akbergenova et al., 2018; Goel et al., 2019; Gratz et al., 2019; Rebola et al., 2019). Previous work showed that the number of release sites *N* changes according to the applied stimuli the specific synapse faces (Atwood and Karunanithi, 2002) suggesting that the number of Unc-13^GFSTF^ clusters (equaling *N*) will also change stimulation-dependent. This study implements a newly generated and highly suitable tool, the Unc-13^GFSTF^-tag MiMIC line, to address this question of structure-function relationship in future studies. We show that Unc-13^GFSTF^ expression at the *Drosophila* NMJ is heterogeneous and varies between individual AZs (Figure 4). This finding matches the heterogeneity of synaptic release and, as the amount of other crucial AZ components like Brp seems to correlate with the amount of evoked release, creating release maps of Unc-13^GFSTF^ can be an appealing experimental approach to clarify the functional relevance of distinct nano-arrangements (Peled et al., 2014; Akbergenova et al., 2018; Newman et al., 2022). In this study, we tagged both Unc-13 isoforms Unc-13A and Unc-13B, however, different expression patterns and roles in AZ assembly have been described for these isoforms (Piao and Sigrist, 2022). Relating isoform-specific Unc-13 imaging and our findings is attractive but beyond the scope of this manuscript. Nevertheless, data distribution might point toward relative contributions of the isoforms to the decreased radial distance of Unc-13^GFSTF^ SCs during PHP. The median changes by ∼5.1%, while 25th and 75th percentiles change by ∼3.6 and ∼4.6%, respectively. This indicates a relatively higher change in the part of the distribution that is far from AZ centers, possibly corresponding to Unc-13B. The Unc-13A N-terminus has been shown to be essential for expression of PHP at the *Drosophila* AZ (Böhme et al., 2019). We tested that insertion of the GFSTF-tag into the *Drosophila unc-13* gene leading to visualization of all Unc-13 isoforms does not disturb the ability of the NMJ to undergo functional PHP (Figure 3E). Specific antibodies for visualization of the Unc-13A N- and C-terminus have been published (Reddy-Alla et al., 2017; compare Figure 1C) with our Unc-13^GFSTF^ tag lying in between. Assuming localization of the N-terminal epitope close to presynaptic Ca^2+^ channels co-staining of the N-terminal antibody and Unc-13^GFSTF^ might allow visualization of the Unc-13 orientation at the presynaptic AZ. We show co-localization of all Unc-13 isoforms using the GFSTF-tag with Brp. Applying HDBSCAN cluster analysis allows precise determination of the Unc-13^GFSTF^ nanoarrangement within presynaptic AZs. We used PhTx for induction of acute PHP to investigate the effect on Unc-13^GFSTF^. We previously found compaction of Brp and RBP within the AZ in models of acute and chronic PHP (Mrestani et al., 2021), thus, we hypothesized that Unc-13^GFSTF^ nanoarchitecture might also change. Indeed, we found a decreased radial distance of Unc-13^GFSTF^ SCs in acute PHP accompanied by a smaller extent of the Unc-13^GFSTF^ area per entire AZ (Figure 4E). These changes reveal compaction of Unc-13 at the AZ during an acute homeostatic challenge reflecting perhaps the functional compensation of enhanced neurotransmitter release after disturbance of postsynaptic glutamate receptors (compare Mrestani et al., 2021). The reported structural changes are small, however, earlier data simulation approaches showed that moderate changes in 2D localization data translate into larger changes in 3D molecule configuration (Mrestani et al., 2021). Future studies investigating the co-localization of Unc-13^GFSTF^ with other AZ epitopes e.g., RBP, VGCCs or RIM appear attractive to decipher the AZ nanoarchitecture further. Especially in the case of less abundant proteins such as VGCCs these imaging approaches are demanding. Our labeling strategy offers the possibility to co-stain different proteins with the same multi-tag GFSTF creating similar imaging conditions. Here, we used the EGFP-tag within Unc-13^GFSTF^ to visualize Unc-13 *via* a secondary Alexa Fluor647-labeled F(ab’)_2_ fragment. Further work using nanobody-staining to visualize the EGFP-tag or labeling strategies detecting the Strep-, FlaSH-tag, or TEV-tag within the GFTSF-multi-tag are conceivable.

Combining the Unc-13^GFSTF^ MiMIC line with knock-out strains of crucial AZ proteins such as RIM or RBP to elucidate their effects on the amount or expression pattern of Unc-13 appears promising. Due to the interaction of Munc-13, Rab3, and RIM in mammals (Brockmann et al., 2020) a RIM knock-out is likely to affect the Munc-13 nanotopology, however, this is unclear in *Drosophila* as formation of the tripartite complex is lacking. The RBP knock-out should affect Unc-13^GFSTF^ expression in the fly (Petzoldt et al., 2020) which can be addressed using the here described MiMIC line. In conclusion, this genetic tool should be useful for further studies analyzing AZ nanoarchitecture and structure-function relationships at the *Drosophila* NMJ.

## Data availability statement

The original contributions presented in this study are included in the article/Supplementary material, further inquiries can be directed to the corresponding authors.

## Author contributions

SD, AM, MH, and MMP designed experiments. SD, AM, FG, MP, FK, and MMP performed experiments. SD, AM, FG, MP, PK, MS, and MMP analyzed the data. SD, AM, and MMP wrote the manuscript with help of all co-authors. MH and MMP coordinated the study and provided funding. All authors contributed to the article and approved the submitted version.

## Footnotes

1
https://www.biozentrum.uni-wuerzburg.de/super-resolution/startseite/

2
https://www.python.org/

3
https://jupyter.org/index.html

4
https://github.com/scikit-learn-contrib/hdbscan

5
https://www.cgal.org
