# Supplementary material for: Endogenous tagging of Unc-13 reveals nanoscale reorganization at active zones during presynaptic homeostatic potentiation
Source: Front Cell Neurosci. 2022 Dec 14;16:1074304. doi: 10.3389/fncel.2022.1074304 (PMC9797049; doi:10.3389/fncel.2022.1074304)
Supplement: Supplementary file 1 [file Data_Sheet_1.docx]

**TITLE**

Endogenous tagging of Unc-13 reveals nanoscale reorganization at active zones during presynaptic homeostatic potentiation

**AUTHORS**

Sven Dannhäuser^1,*^, Achmed Mrestani^1,2,3*^, Florian Gundelach^1,4^, Martin Pauli^1^, Fabian Komma^1^, Philip Kollmannsberger^5^, Markus Sauer^6^, Manfred Heckmann^1,#^, Mila M. Paul^1,7,#^

**SUPPLEMENTARY MATERIAL**

- 2 Supplementary Figures
- 4 Supplementary Tables

**
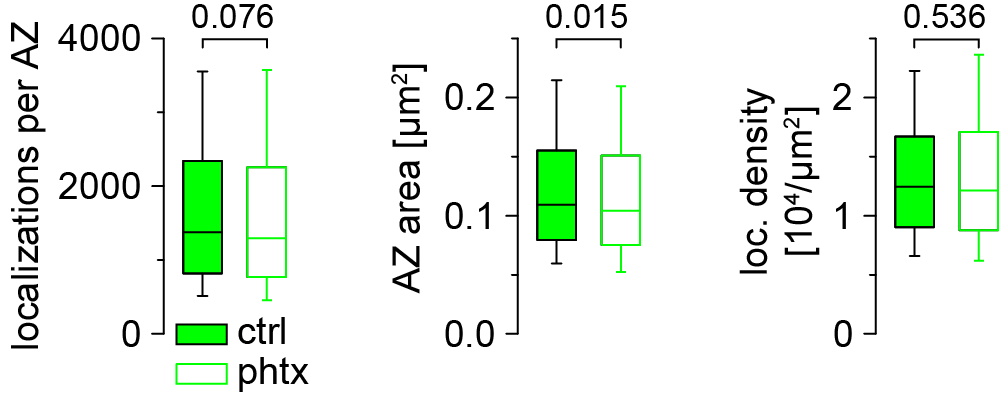
**

**Supplementary Figure 1. Active zone compaction using the dye Alexa Fluor532.**

Number of Brp localizations per AZ, AZ area and Brp localization density for ctrl (filled boxes, n = 1,462 AZs from 22 NMJs and 9 animals) and phtx (open boxes, n = 1,521 AZs from 23 NMJs and 9 animals) shown as box plots (horizontal lines show median, boxes boundaries 25^th^ and 75^th^ percentiles and whiskers 10^th^ and 90^th^ percentiles) imaged in *unc-13^GFSTF^* type Ib boutons stained with Brp^Nc82^ labelled with Alexa Fluor532 conjugated IgGs (green channel from data in Figure 4).


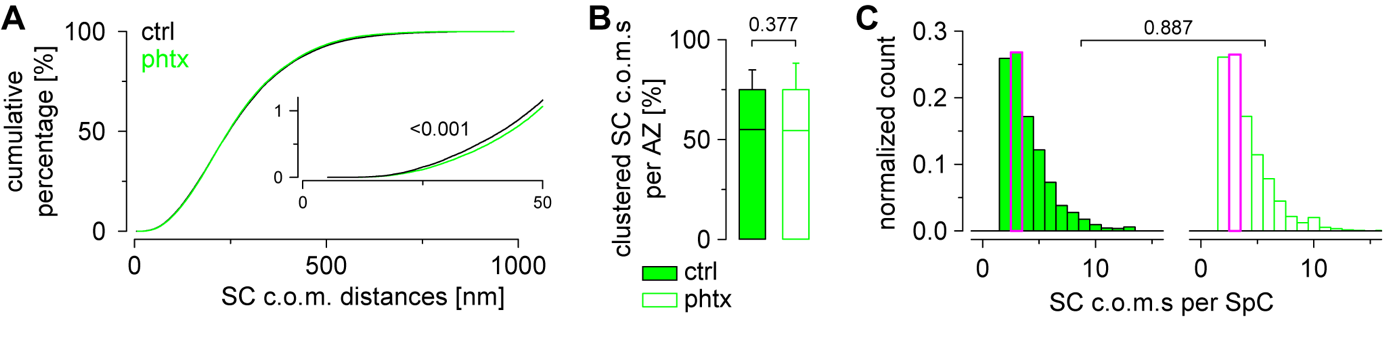


**Supplementary Figure 2. Extended Unc-13 supercluster analysis.**

(A) Cumulative plots show distance distributions between Unc-13^GFSTF^ SC c.o.m.s at circular AZs for ctrl (n = 162,170 distances from 10,461 SCs and 876 AZs) and phtx (n = 169,874 distances from 10,988 SCs and 927 AZs). Inset shows x and y axes from 0 to 50 nm and 0 to 1.2 %, respectively.

(B) Percentage of SC c.o.m.s that are organized in SpCs per AZ for ctrl (n = 876 AZs from 22 NMJs and 9 animals) and phtx (n = 927 AZs from 23 NMJs and 9 animals).

(C) Number of SC c.o.m.s per SpC for both groups at AZs with at least one SpC (n = 1,417 and 1,497 SpCs for ctrl and phtx, respectively).

| **parameter** | **wt** | ***unc-13^GFSTF^*** | **p** |
| --- | --- | --- | --- |
| **mEPSC amplitude**  **[-nA]** | 0.713 ± 0.032  n = 13 NMJs, 7 larvae | 0.681 ± 0.023  n = 13 NMJs, 7 larvae | 0.428 |
| **mEPSC frequency**  **[ms]** | 2.038 ± 0.168  n = 13 NMJs, 7 larvae | 2.242 ± 0.243  n = 13 NMJs, 7 larvae | 0.496 |
| **eEPSC amplitude**  **[-nA]** | 44.260 ± 3.116  n = 14 NMJs, 8 larvae | 43.350 ± 2.204  n = 14 NMJs, 7 larvae | 0.8743 |
| **quantal content** | 87.900 ± 8.282  n = 13 NMJs 7 larvae | 84.990 ± 3.628  n = 13 NMJs, 7 larvae | 0.7503 |
| **paired-pulse ratio**  **10 ms**  **30 ms**  **100 ms**  **300 ms**  **1000 ms** | 1.233 ± 0.023  1.187 ± 0.021  1.078 ± 0.014  1.015 ± 0.021  0.999 ± 0.017  n = 10 NMJs, 6 larvae | 1.181 ± 0.019  1.165 ± 0.043  1.059 ± 0.019  1.010 ± 0.017  0.987 ± 0.013  n = 10 NMJs, 6 larvae | 0.091  0.643  0.405  0.875  0.572 |

**Supplementary Table 1. Electrophysiological analysis of spontaneous and evoked synaptic transmission in *unc-13^GFSTF^* and wildtype larvae. Related to Figure 3A-D.** Numerical values are given as mean ± SEM for each genotype. p-values and sample sizes for the number of NMJs and the number of animals used for analysis are indicated. Data were normally distributed and unpaired t-tests were used to evaluate statistical significance.

| **parameter** | **wt (DMSO)** | **wt (PhTx)** | ***unc-13^GFSTF^***  **(DMSO)** | ***unc-13^GFSTF^***  **(PhTx)** |
| --- | --- | --- | --- | --- |
| **eEPSC amplitude**  **[-nA]** | 37.83 ± 2.45  n = 10 NMJs, 7 larvae | 44.20 ± 2.31  n = 10 NMJs, 5 larvae | 41.99 ± 2.26  n = 13 NMJs, 6 larvae | 47.14 ± 2.88  n = 14 NMJs, 7 larvae |
| **mEPSC amplitude**  **[-nA]** | 0.647 ± 0.065  n = 10 NMJs, 7 larvae | 0.413 ± 0.020  n = 10 NMJs, 5 larvae | 0.716 ± 0.033  n = 13 NMJs, 6 larvae | 0.393 ± 0.017, n = 14 NMJs, 7 larvae |
| **quantal content** | 84.89 ± 9.82  n = 10 NMJs, 7 larvae | 144.90 ± 9.03  n = 10 NMJs, 5 larvae | 80.25 ± 5.47  n = 13 NMJs, 6 larvae | 165.50 ± 14.02, n = 14 NMJs, 7 larvae |

**Supplementary Table 2. Electrophysiological analysis of acute presynaptic homeostasis in *unc-13^GFSTF^* and wildtype animals. Related to Figure 3E.** Numerical values are given as mean ± SEM for each group. p-values and sample sizes for the number of NMJs and the number of animals used for analysis are indicated. For information of statistical analysis see Supplementary Table 3.

| **genotype** | **eEPSC amplitude** | **mEPSC amplitude** | **quantal content** |
| --- | --- | --- | --- |
| wt (DMSO)  vs. wt (PhTx)  vs. *unc-13^GFSTF^* (DMSO) | 0.075  0.229 | 0.002  0.257 | 0.001  0.784 |
| wt (PhTx)  vs. *unc-13^GFSTF^* (PhTx) | 0.462 | 0.472 | 0.472 |
| *unc-13^GFSTF^* (DMSO)  vs. *unc-13^GFSTF^* (PhTx) | 0.176 | < 0.0001 | < 0.0001 |

**Supplementary Table 3. Statistical comparison of acute presynaptic homeostasis in *unc-13^GFSTF^* and wildtype animals. Related to Figure 3E.** p-values revealed by parametric t-tests (eEPSC amplitude) or by Mann-Whitney Rank Sum tests for non-parametric data (mEPSC amplitude and quantal content) are given for comparisons between both genotypes or between measurements in DMSO and PhTx within an individual genotype.

| **parameter** | **ctrl** | **phtx** | **p-value** |
| --- | --- | --- | --- |
| **Unc-13^GFSTF^** | | | |
| locs. per SC | 18 (11-32) | 18 (11-31) | 0.062 |
| SC area [nm^2^] | 524 (220-1,128) | 528 (232-1,088) | 0.443 |
| SC loc. density [locs./µm^2^] | 36,741 (24,732-61,297) | 36,106 (24,566-58,453) | 0.002 |
| n (SCs, NMJs, animals) | 20,037, 22, 9 | 20,393, 23, 9 |  |
| SCs per AZ | 11 (7-19) | 11 (7-18) | 0.329 |
| locs.per AZ | 306 (199-480) | 293 (179-465) | 0.012 |
| area per AZ [nm^2^] | 10,589 (6,797-15,820) | 9,923 (6,423-15,616) | 0.023 |
| loc. density per AZ [locs./µm^2^] | 29,933 (24,611-35,606) | 29,987 (24,797-36,054) | 0.960 |
| radial distance [nm] | 136 (110-174) | 129 (106-166) | < 0.001 |
| n (AZs, NMJs, animals) | 1,462, 22, 9 | 1,521, 23, 9 |  |
| SC c.o.m. distances [nm] | 170 (103-256) | 173 (105-257) | < 0.001 |
| n (distances, SCs, AZs) | 162,170, 10,461, 876 | 169,874, 10,988, 927 |  |
| nearest neighbor distance [nm] | 50 (30-72) | 51 (32-72) | 0.003 |
| n (distances, AZs) | 10,461, 876 | 10,988, 927 |  |
| second neighbor distance [nm] | 71 (48-97) | 72 (51-97) | 0.015 |
| n (distances, AZs) | 10,425, 858 | 10,940, 903 |  |
| third neighbor distance [nm] | 88 (64-121) | 90 (67-121) | < 0.001 |
| n (distances, AZs) | 10,329, 826 | 10,856, 875 |  |
| fourth neighbor distance [nm] | 105 (77-143) | 107 (81-145) | < 0.001 |
| n (distances, AZs) | 10,185, 790 | 10,688, 833 |  |
| fifth neighbor distance [nm] | 120 (90-165) | 123 (93-166) | < 0.001 |
| n (distances, AZs) | 9,945, 742 | 10,493, 794 |  |
| clustered SC c.o.m.s per AZ [%] | 55.1 (0.0-75.0) | 54.5 (0.0-75.0) | 0.377 |
| n (AZs, NMJs, animals) | 876, 22, 9 | 927, 23, 9 |  |
| SpCs per AZ | 2 (2-3) | 2 (2-3) | 0.234 |
| n (AZs, NMJs, animals) | 501, 22, 9 | 543, 23, 9 |  |
| SC c.o.m.s per SpC | 3 (2-5) | 3 (2-5) | 0.887 |
| SC c.o.m. to SpC c.o.m. distance [nm] | 35 (25-47) | 37 (27-50) | 0.005 |
| n (SpCs) | 1,417 | 1,497 |  |
| **Brp Alexa Fluor532** | | | |
| localizations per AZ | 1,375 (818-2,342) | 1,296 (769-2,260) | 0.076 |
| AZ area [µm^2^] | 0.109 (0.08-0.155) | 0.104 (0.075-0.151) | 0.015 |
| AZ loc. density [locs./µm^2^] | 12,467 (9035-16,705) | 12,140 (8,773-17,093) | 0.536 |
| circularity [a.u.] | 0.66 (0.51-0.78) | 0.67 (0.53-0-79) | 0.124 |
| n (AZs, NMJs, animals) | 1,462, 22, 9 | 1,521, 23, 9 |  |

**Supplementary Table 4. *d*STORM analysis of Unc-13^GFSTF^ subclusters using Alexa Fluor647 and Brp clusters using Alexa Fluor532. Related to Figures 4 and 5 and Supplementary Figure 2.** Non-parametric data, reported as median (25^th^-75^th^ percentile).
